# The deubiquitinase USP40 preserves endothelial integrity by targeting the heat shock protein HSP90β

**DOI:** 10.1038/s12276-024-01160-y

**Published:** 2024-02-02

**Authors:** Jiaxing Miao, Lian Li, Nargis Shaheen, Jianxin Wei, Anastasia M. Jacko, Prithu Sundd, Sarah J. Taleb, Rama K. Mallampalli, Yutong Zhao, Jing Zhao

**Affiliations:** 1 Department of Physiology and Cell Biology, Dorothy M. Davis Heart and Lung Research Institute, Columbus, OH USA; 2 Department of Medicine, The University of Pittsburgh, Pittsburgh, PA USA; 3 Department of Internal Medicine, The Ohio State University, Columbus, OH USA

**Keywords:** Translational research, Deubiquitylating enzymes

## Abstract

Endothelial cell (EC) barrier disruption and inflammation are the pathological hallmarks of vascular disorders and acute infectious diseases and related conditions, including the coronavirus disease 2019 (COVID-19) and sepsis. Ubiquitination plays a critical role in regulating the stability, intracellular trafficking, and enzymatic activity of proteins and is reversed by deubiquitinating enzymes (DUBs). The role of DUBs in endothelial biology is largely unknown. In this study, we report that USP40, a poorly characterized DUB, prevents EC barrier disruption through reductions in the activation of RhoA and phosphorylation of myosin light chain (MLC) and cofilin. Furthermore, USP40 reduces EC inflammation through the attenuation of NF-ĸB activation, ICAM1 expression, and leukocyte-EC adhesion. We further show that USP40 activity and expression are reduced in response to endotoxin challenge. Global depletion of USP40 and EC-targeted USP40 depletion in mice exacerbated experimental lung injury, whereas lentiviral gene transfer of USP40 protected against endotoxin-induced lung injury. Using an unbiased approach, we discovered that the protective effect of USP40 occurs through the targeting of heat shock protein 90β (HSP90β) for its deubiquitination and inactivation. Together, these data reveal a critical protective role of USP40 in vascular injury, identifying a unique mechanistic pathway that profoundly impacts endothelial function via DUBs.

## USP40: a key player in preventing vascular injury and inflammation

Vascular endothelial cells (ECs - cells that line the interior of blood vessels) play a vital role in maintaining the health of blood vessels and controlling inflammation. Damage or malfunction of these cells can lead to severe inflammatory diseases like acute lung injury and sepsis. The molecular mechanisms (processes at a molecular level) that control these cells’ function are not completely understood. In this research, a new protein, USP40, was identified that helps safeguard ECs from inflammation and maintains their barrier function. This protein functions by deubiquitinating (removing ubiquitin, a small protein) and inactivating another protein, HSP90, thereby reducing its ability to instigate inflammation and disrupt the EC barrier. These results suggest that USP40 could be a potential treatment target for inflammatory diseases.

This summary was initially drafted using artificial intelligence, then revised and fact-checked by the author.

## Introduction

Vascular ECs maintain vessel integrity. EC hyperpermeability and inflammation are important pathogenic features of inflammatory diseases such as acute lung injury and sepsis^1,2^. Loss of endothelial barrier integrity leads to an influx of protein-rich edema fluid into the interstitial tissue^2,3^. Stress fibers are formed by contractile actin-myosin bundles in ECs under inflammatory conditions such as bacterial or viral infection^3–5^. Contractile forces disrupt cell‒cell junctions and increase EC permeability^6,7^. RhoA, a small GTPase, plays a central role in stress fiber assembly and EC hyperpermeability by regulating two pathways^6,8^. Activation of RhoA triggers the phosphorylation of myosin light chain (MLC), leading to stress fiber formation. Additionally, RhoA-dependent kinase (ROCK) activates LIMK1 (LIM domain kinase 1), resulting in the phosphorylation and inactivation of cofilin, an actin depolymerizing factor, thereby stabilizing stress fibers and promoting EC barrier disruption^9,10^. EC inflammation entails EC dysfunction and tissue damage by promoting neutrophil infiltration to the site of inflammation^11^. Increased expression of adhesion molecules, such as ICAM1, on the EC surface is required for neutrophil adhesion to ECs and transmigration across the endothelial barrier^12,13^. Proinflammatory stimuli induce ICAM1 expression in ECs through the NF-κB pathway, which is activated by IKKβ-mediated phosphorylation of I-κB^14,15^.

HSP90, a chaperone protein, activates RhoA. Inhibition of Hsp90 prevents LPS-induced endothelial barrier disruption by attenuating RhoA signaling^16^. Moreover, inhibition of HSP90 decreases NF-ĸB activation in response to LPS or tumor necrosis factor α (TNFα) stimulation^17,18^. Thus, HSP90 plays a central role in the regulation of EC barrier disruption and inflammation^16^. Understanding the molecular regulation of HSP90 activation is important for the development of a therapeutic strategy to maintain endothelial function. Acetylation of HSP90 results in a reduction in its activity and its dissociation from its binding partners, thus suppressing HSP90-mediated biological functions^19,20^. Ubiquitination is a major posttranslational modification involved in protein localization, protein‒protein interactions, and enzymatic activity^21,22^. Ubiquitin E3 ligases mediate ubiquitination, which can be reversed by DUBs. The ubiquitin E3 ligases CHIP and Hectd1 mediate HSP90 ubiquitination^23,24^; however, a DUB for HSP90 has not been identified.

USP40, a newly recognized DUB, has been shown to regulate glomerular permeability in zebrafish. USP40 gene knockdown disrupts glomerular barrier integrity by reducing the expression of nestin, a filament protein^25^. However, a study from the same group demonstrated that nestin expression was upregulated in the glomeruli of USP40KO mice. However, unlike in zebrafish, no apparent phenotypic changes were observed in USP40KO mice^25,26^. These contrasting findings indicate that the molecular mechanism of USP40-mediated EC biological functions differs across species. Through an unbiased screening approach, we found that USP40 attenuates LPS- or thrombin-induced human lung microvascular endothelial cell (HLMVEC) barrier disruption and LPS-induced EC inflammatory responses, such as ICAM1 expression and neutrophil-EC interactions. USP40 exhibits protective effects on ECs, but our findings indicate that the underlying molecular mechanisms differ from those found in a previous study^25^. No reduction in nestin expression was found in USP40-deficient HLMVECs or in the lungs of USP40KO mice. Furthermore, our data reveal that USP40 deubiquitinates HSP90, thereby increasing its acetylation and reducing its activation. Our observations are the first to reveal that USP40 reduces the severity of acute lung injury by reducing HSP90 ubiquitination, thereby attenuating the activation of RhoA and NF-κB, EC permeability, and inflammation.

## Materials and methods

### Cell culture and reagents

Human lung microvascular endothelial cells (HLMVECs), THP-1 cells, MLE12 cells, and Raw 264.7 cells were purchased from American Type Culture Collection (ATCC, Manassas, VA, USA). HLMVECs were cultured with EGM-2MV microvascular endothelial growth medium containing 5% fetal bovine serum (FBS) at 37 °C in 5% CO_2_. LPS (*E. coli* O55:B5), thrombin, and the anti-β-actin antibody were purchased from Sigma–Aldrich (St. Louis, MO, USA). Antibodies against VE-cadherin, USP40, ICAM1, VCAM1, Lamin A/C, and GAPDH and immobilized protein A/G beads were purchased from Santa Cruz Biotechnology (Dallas, TX, USA). Antibodies against pMLC, MLC, the Flag tag, the V5 tag, pLimk1, Limk1, p-cofilin 1, cofilin 1, pIκBα, p65, p-p65, HSP90β, K63-linked ubiquitin, ubiquitin, and acetylated lysine (AcK) were purchased from Cell Signaling (Beverly, MA, USA). ELISA kits for quantifying TNFα, IL-6, and IL-8 were obtained from eBioscience (San Diego, CA), and mouse IL-1β, KC/CXCL1 ELISA kits, and human recombinant TNFα protein were purchased from R&D Systems (Minneapolis, MN, USA). The anti-CD31-FITC antibody was obtained from Biolegend (San Diego, CA, USA). GeneJet™ reagent and GeneMute siRNA transfection reagent were purchased from SignaGen (Frederick, MD, USA). All materials used in the experiments were of the highest grade commercially available.

### Animals and LPS administration

Male and female C57BL/6 J mice were housed and cared for in the specific pathogen-free animal care facility at The Ohio State University in accordance with institutional guidelines and the guidelines of the US National Institutes of Health. All animal experiments were approved by the Animal Care and Use Committee at The Ohio State University and were performed in accordance with the guidelines outlined by the committee. The CRISPR/Cas9 system was used by the Innovative Technologies Development Core Facility at the University of Pittsburgh to generate USP40 global knockout (USP40^−/−^) and USP40-Loxp mice^27,28^. USP40 EC knockout mice were generated by crossing USP40floxp/floxp mice with Tek-Cre or Cdh5-Cre transgenic mice (B6.Cg-Tg^Tek-Cre1Ywa/J^ or B6.Cg-Tg^Cdh5-cre7Mlia/J^, respectively; The Jackson Laboratory). Lung injury models were established by intratracheal administration of LPS (2 mg/kg body weight) for 24 h. For construction of the lentiviral vector delivery system, human USP40 cDNA was inserted into the pLVX-IRES-tdTomato vector (Clontech, Palo Alto, CA, USA). C57BL/6 J mice were intravenously administered lentiviral vectors or lenti-USP40 (5 × 10^7^ plaque-forming units per mouse) for 7 days before intratracheal injection of LPS. BALF and lung tissues were collected for further evaluation by ELISA, western blotting, H&E staining, wet/dry ratio measurement, immunohistochemical staining, and immunofluorescence staining.

### Cecal ligation and puncture (CLP)-induced polymicrobial sepsis

Mice were anesthetized by intraperitoneal injection of ketamine (90 mg/kg) and xylazine (10 mg/kg). A 3-cm longitudinal incision was made in the lower abdomen; the cecum with the adjoining intestine was then externalized and ligated 0.5 cm from its end with a 3.0 silk suture. Then, the ligated cecum was punctured with an 18-gauge needle, allowing entrapped fecal material to leak into the normally sterile peritoneal cavity. The cecum was then repositioned in the peritoneal cavity, and the abdomen was closed. Sham-operated animals received laparotomy only.

### Flow cytometric analysis of mouse lung cells

Isolated single lung cells were incubated with a rat anti-mouse CD16/32 antibody (Biolegend) for 30 min to block Fc receptors. Fluorophore-conjugated antibodies were added at the recommended dilutions, and the cells were then incubated with the specific antibodies in the dark for an additional 30 min. The following anti-mouse antibodies were used for cell surface marker staining: anti-CD31-PE/Cy7 (endothelial cell marker), anti-CD54-PE (a surrogate marker of antigen-presenting cell activation), anti-CD11c-FITC, and anti-Ly6G-APC/Cy7 (expressed on neutrophils) antibodies purchased from Biolegend (San Diego, CA); an anti-CD326-APC (epithelial cell surface marker) antibody purchased from eBioscience (San Diego, CA); and an anti-CD45-Percp (leukocyte common antigen) antibody purchased from BD Bioscience (San Jose, CA). Data acquisition and data analysis were performed with an Agilent NovoCyte flow cytometer and NovoExpress Software, respectively. We defined cell compartments as follows: endothelial, CD326^−^ CD45^−^/CD31^+^; and neutrophil, CD45+/Ly6G+.

### Measurement of TEER (transendothelial electrical resistance) by an electrical cell-substrate impedance sensing system (ECIS)

HLMVECs were grown on gold electrodes. Resistance changes were monitored in real time using the ECIS (Applied Biophysics) at 4000 Hz. TEER values for each microelectrode were pooled at discrete time points and plotted against time as the mean ± S.E.M. values.

### Immunoblot analysis

HLMVECs and lung tissues were lysed in cell lysis buffer containing 20 mM Tris HCl (pH 7.4), 150 mM NaCl, 2 mM EGTA, 5 mM β-glycerophosphate, 1 mM MgCl_2_, 1% Triton X-100, 1 mM sodium orthovanadate, 10 μg/ml protease inhibitors, 1 μg/ml leupeptin, and 1 μg/ml pepstatin. Equal amounts of protein were separated by SDS‒PAGE. Immunoblotting with primary and secondary antibodies was performed as described above^29^. Signals on the membrane were detected by an Azure c600 imaging system. The intensities of the bands were determined by ImageJ software.

### Ubiquitination assay

Cells were harvested in cold PBS. Cell pellets were suspended in 50–80 μl of 2% SDS lysis buffer with 1 μl of ubiquitin aldehyde and 1 μl of NEM. After sonication, the cell lysates were boiled at 100 °C for 10 min. The samples were diluted with 500–800 μl of 1× Tris-buffered saline and subjected to immunoprecipitation (IP) with an anti-HSP90β antibody followed by an anti-ubiquitin or anti-K63-linked ubiquitin antibody.

### DUB activity assay

Cells transfected with empty vector or the Flag-USP40 plasmid were collected, and Flag-USP40 was immunoprecipitated with FLAG-M2 affinity gel (Sigma). The beads were resuspended in DUB assay buffer (40 mM Tris (pH 7.1), 100 mM NaCl, and 5 mM DTT) and divided into 10 µl aliquots after washing with TBS buffer containing 0.1% Triton X-100. Ten microliters of Flag-USP40-conjugated beads and 2 µg of purified K63-linked or K48-linked di-Ub in 40 µl of reaction buffer were incubated at 37 °C for 2 h with gentle mixing. The DUB cleavage products of K63-linked or K48-linked di-Ub were visualized by immunoblotting with an anti-ubiquitin antibody after the reactions were terminated with SDS sample buffer. DUB activity was continuously monitored by incubation of Flag-USP40 beads with Ubiquitin-AMC (UBPBio, Inc., Aurora, CO, USA), a fluorogenic substrate. DUB activity was determined using fluorescence spectrophotometry with an excitation wavelength of 360 and an emission wavelength of 460 nm.

### ATPase activity assay

Cells cotransfected with HSP90-HA and Flag-USP40 or empty vector were collected, and HA-HSP90 was immunoprecipitated with HA affinity gel (Sigma). Hsp90 ATPase activity was determined by a Transcreener ADP² Assay (BellBrook Labs, Madison WI, USA), a direct method for measuring the amount of ADP generated during a kinase reaction. Fluorescence polarization (FP) measurement with Alexa Fluor® 633 was performed on a CLARIOstar Plus plate reader.

### Proteomic analysis of USP40-interacting proteins by immunoprecipitation and mass spectrometry

USP40-interacting proteins were immunoprecipitated from Flag-USP40-transfected HLMVECs using FLAG-M2 affinity gel (Sigma). For proteomic analysis, the USP40-interacting proteins were eluted from the beads under reducing conditions, separated by SDS‒PAGE, and stained with Coomassie blue. The protein bands were excised, and in-gel trypsin digestion was performed as described previously^30^. LC‒MS analysis was performed at the core facility of the University of Pittsburgh.

### Statistical analysis

All data were subjected to statistical analysis using one-way or two-way ANOVA followed by Tukey’s post hoc test or unpaired Student’s t test to compare continuous variables or the Mantel‒Cox (log-rank) test to compare survival curves. Data are expressed as the mean ± SEM of triplicate samples from at least three independent experiments. Values of *p* < 0.05 were considered statistically significant.

## Results

### USP40 plays a protective role in EC barrier function

**Fig. 1:**
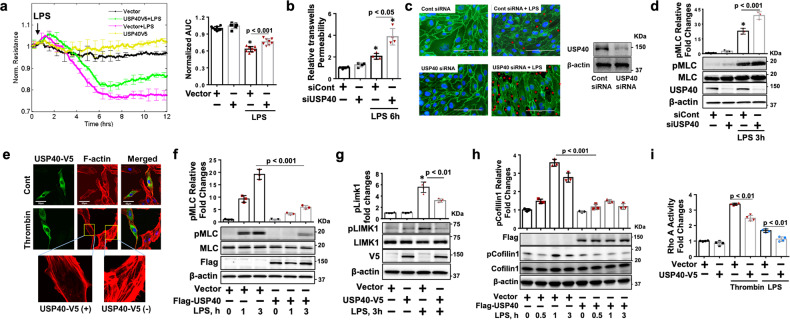
USP40 preserves lung EC barrier integrity. **a** HLMVECs were transfected with empty vector or the USP40-V5 plasmid for 48 h and were then treated with LPS (0.2 μg/ml). TEER was measured by the ECIS system. Normalized resistance was recorded at 4000 Hz. Each line indicates the mean ± SEM at the specified time points. The area under the curve (AUC) bar plots show the quantification of the AUCs for changes in barrier resistance. *: Significant difference (*p* < 0.001) versus the values in untreated Vector and USP40-V5 cells as well as LPS-treated USP40-V5 cells. **b** HLMVECs grown in transwell inserts (0.4 μm) were transfected with control siRNA (siCont) or USP40 siRNA (siUSP40) for 72 h and then treated with LPS for 6 h. Leakage of FITC-labeled dextran was measured and quantified. *: Significant difference (*p* < 0.001) versus the values in untreated siCont and siUSP40 cells and (*p* < 0.05) between LPS-treated siCont and siUSP40 cells, as determined by one-way ANOVA. **c** HLMVECs grown on glass-bottom dishes were transfected with control siRNA or USP40 siRNA for 72 h and then treated with LPS for 6 h. Cells were subjected to immunofluorescence staining with an anti-VE-cadherin antibody (green). Nuclei were stained with DAPI (blue). The red arrows indicate gap formation. Scale bars = 100 µm. The effect of USP40 siRNA was confirmed by immunoblotting. **d** HLMVECs were transfected with control siRNA or USP40 siRNA for 72 h and then treated with LPS (0.2 μg/ml) for 3 h. Immunoblot analysis was performed with the indicated antibodies. Quantification of the pMLC protein level relative to the MLC level was performed by densitometry. *: Significant difference (*p* < 0.001) versus the values in untreated siCont and siUSP40 cells as well as LPS-treated siUSP40 cells, as determined by one-way ANOVA with Tukey’s multiple comparison test. **e** HLMVECs grown on glass-bottom dishes were transfected with empty vector or USP40-V5 for 48 h and then treated with thrombin (1 U/ml) for 0.5 h. F-actin and stress fibers were fluorescently stained with phalloidin. Scale bars = 50 µm. **f** HLMVECs were transfected with empty vector or Flag-USP40 for 48 h and then treated with LPS (0.2 μg/ml) for 1 and 3 h. Immunoblot analysis was performed with the indicated antibodies. Quantification of the pMLC protein level relative to the MLC level was performed by densitometry (*n* = 3). Significant differences (*p* < 0.001) between the two groups were determined by two-way ANOVA. **g** HLMVECs were transfected with empty vector or USP40-V5 for 48 h and then treated with LPS (0.2 μg/ml) for 3 h. Immunoblot analysis was performed with the indicated antibodies. Quantification of the pLIMK1 protein level relative to the LIMK1 level was performed by densitometry. *: Significant difference (*p* < 0.001) versus the values in untreated Vector and USP40-V5 cells and (*p* < 0.01) between LPS-treated Vector and USP40-V5 cells, as determined by one-way ANOVA with Tukey’s multiple comparison test. **h** HLMVECs were transfected with empty vector or Flag-USP40 for 48 h and then treated with LPS (0.2 μg/ml) for 0.5–3 h. Immunoblot analysis was performed with the indicated antibodies. Quantification of the pCofilin1 protein level relative to the cofilin 1 level was performed by densitometry. Fold changes in the pCofilin 1 level were analyzed. Significant differences (*p* < 0.001) between the two groups were determined by two-way ANOVA. **i** HLMVECs were transfected with empty vector or USP40-V5 for 48 h and then treated with thrombin (1 U/ml, 0.5 h) or LPS (0.2 μg/ml, 1 h). RhoA activity was measured and quantified according to the manufacturer’s instructions.

DUBs negatively regulate ubiquitination, thus modulating protein stability or enzyme activity. The effect of DUBs on endothelial barrier integrity has not been well studied. To identify which DUB affects endothelial barrier function, more than 15 plasmids encoding different DUBs were transfected into HLMVECs, and the cells were then treated with thrombin. Phosphorylation of MLC triggers actin stress fiber formation, resulting in EC contraction and hyperpermeability. Among the overexpressed DUBs, only USP40 attenuated thrombin-induced phosphorylation of MLC (Supplementary Fig. 1a). Transendothelial resistance (TEER) was measured using the ECIS system. Overexpression of USP40 attenuated the decreases in TEER induced by LPS (Fig. 1a) and thrombin (Supplementary Fig. 1b). Downregulation of USP40 exacerbated LPS-induced permeability (Fig. 1b) and intercellular gap formation (Fig. 1c), as well as LPS- and TNFα-induced phosphorylation of MLC (Fig. 1d, Supplementary Fig. 1c) in HLMVECs. The specificity of the USP40 siRNA was confirmed as shown in Supplementary Fig. 1f. Overexpression of USP40 attenuated stress fiber formation induced by thrombin (Fig. 1e), LPS and TNFα (Supplementary Fig. 1d). LPS treatment of HLMVECs increased the phosphorylation of MLC, while it was reduced by USP40-V5 overexpression (Fig. 1f), suggesting that USP40 negatively regulates the phosphorylation of MLC. LIMK1-mediated phosphorylation of cofilin reduces actin filament depolymerization, thus promoting stress fiber stabilization^31^. LPS treatment of HLMVECs induced the phosphorylation of LIMK1 and cofilin 1, while these effects were attenuated by overexpression of USP40 (Fig. 1g, h). Overexpression of USP40 also reduced thrombin-induced phosphorylation of cofilin 1 (Supplementary Fig. 1e), suggesting an inhibitory role of USP40 in stress fiber stabilization. RhoA plays a central role in the regulation of MLC and LIMK1 phosphorylation. To understand the mechanisms by which USP40 inhibits stress fiber formation, RhoA activity was measured. As shown in Fig. 1i, overexpression of USP40 attenuated LPS- and thrombin-induced activation of RhoA, indicating that USP40 protects against proinflammatory agonist-induced EC barrier disruption by preventing RhoA-mediated stress fiber formation and stabilization.

### USP40 attenuates TNFα- and LPS-induced EC inflammation

**Fig. 2:**
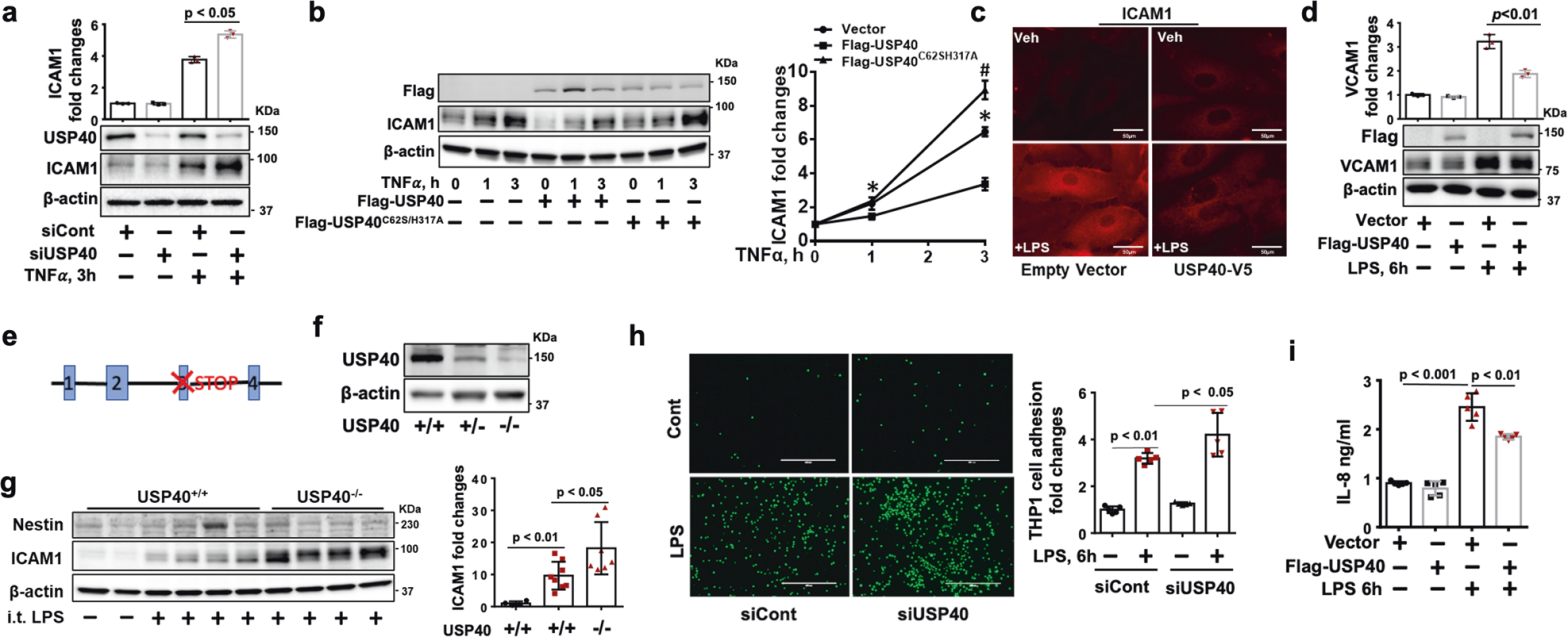
USP40 diminishes EC inflammation. **a** HLMVECs were transfected with control siRNA or USP40 siRNA for 72 h and then treated with TNFα (10 ng/ml) for 3 h. Immunoblot analysis was performed with the indicated antibodies. Fold changes in the ICAM1 protein level relative to the β-actin level were assessed by densitometry (*n* = 3). **b** HLMVECs were transfected with empty vector, Flag-USP40, or Flag-USP40^C62SH317A^ for 48 h and then treated with TNFα (10 ng/ml) for 1–3 h. Immunoblot analysis was performed with the indicated antibodies. Fold changes in the ICAM1 protein level relative to the β-actin level were assessed by densitometry (*n* = 3). *p < 0.01 for Flag-USP40 cells versus TNFα-treated Vector and Flag-USP40^C62SH317A^ cells and ^#^*p* < 0.001 for Flag-USP40^C62SH317A^ cells versus TNFα-treated Vector and Flag-USP40 cells, as determined by two-way ANOVA with Tukey’s multiple comparison test. **c** HLMVECs grown on glass-bottom dishes were transfected with empty vector or the USP40-V5 plasmid and then treated with LPS (0.2 μg/ml) for 6 h. Cells were subjected to immunofluorescence staining with an anti-ICAM1 antibody (red). Scale bars = 50 µm. **d** HLMVECs were transfected with empty vector or the Flag-USP40 plasmid for 48 h and then treated with LPS (0.2 µg/ml) for 6 h. Immunoblot analysis was performed with the indicated antibodies. Fold changes in the VCAM1 protein level relative to the β-actin level were assessed by densitometry (*n* = 3). **e** Mouse *Usp40* exon 3 (chr1: 88003332-88006310) was deleted with the CRISPR/Cas9 system. **f** Immunoblot analysis of lung tissues from wild-type (USP40^+/+^), USP40^+/−^, and USP40^−/−^ mice. **g** USP40^+/+^ and USP40^−/−^ mice were challenged by intratracheal (i.t.) instillation of LPS (4 mg/kg) for 24 h. ICAM1 and nestin levels in lung tissues were analyzed by immunoblotting. Fold changes in the ICAM1/β-actin ratio were analyzed (*n* = 4–8). **h** HLMVECs were transfected with control siRNA or USP40 siRNA for 72 h. Cells were treated with LPS (0.2 μg/ml) for 6 h, and the adhesion of fluorescently labeled THP-1 cells to HLMVECs was then measured by fluorescence microscopy and quantified. **i** HLMVECs were transfected with empty vector or the Flag-USP40 plasmid for 48 h and then treated with LPS (0.2 µg/ml) for 6 h. The IL-8 concentration in the medium was measured by ELISA (*n* = 3–6).

NF-ĸB-driven ICAM1 and VCAM1 expression contributes to neutrophil adhesion to ECs in response to proinflammatory agonists^32^. Downregulation of USP40 increased TNFα-induced ICAM1 expression (Fig. 2a), while overexpression of wild-type USP40 but not an enzyme active site mutant of USP40 (USP40^C625SH317A^) attenuated TNFα-induced ICAM1 expression in HLMVECs (Fig. 2b). LPS increased ICAM1 and VCAM1 expression, and these effects were attenuated in USP40-overexpressing HLMVECs (Fig. 2c, d). In addition to downregulating USP40 by siRNA transfection, we generated USP40-deficient mice (USP40^−/−^) (Fig. 2e). Depletion of USP40 in the lungs was confirmed by immunoblotting (Fig. 2f). Wild-type (USP40^+/+^) and USP40^−/−^ mice were used to establish a murine model of intratracheal LPS-induced acute lung injury. Depletion of USP40 further elevated LPS-induced ICAM1 expression in the lungs without altering nestin expression (Fig. 2g and Supplementary Fig. 2a). ICAM1 and VCAM1 play vital roles in immune cell adherence to the endothelium^33^. Furthermore, we found that downregulation of USP40 in HLMVECs promoted THP-1 cell adherence to HLMVECs (Fig. 2h), while overexpression of USP40 resulted in the opposite effect (Supplementary Fig. 2b). In addition to ICAM1 and VCAM1 expression, USP40 overexpression attenuated LPS-induced IL-8, IL-6, and KC expression in different cell types: HLMVECs, mouse macrophages (RAW264.7), and mouse lung epithelial (MLE12) cells (Fig. 2i, Supplementary Fig. 2c, d). Downregulation of USP40 increased LPS-induced IL-6 secretion by RAW264.7 cells (Supplementary Fig. 2e), indicating that USP40 exhibits anti-inflammatory properties.

### USP40 negatively regulates the NF-κB pathway in HLMVECs

To investigate the effect of USP40 on NF-κB activity, we examined the phosphorylation of I-κBα and the nuclear translocation and phosphorylation of NF-κB65. USP40-V5 overexpression attenuated NF-κBp65 nuclear translocation, p65 phosphorylation, and I-ĸBα phosphorylation in response to TNFα or LPS stimulation (Supplementary Fig. 3a–d). Together, these findings indicate that USP40 negatively regulates the NF-κB pathway.

### USP40 activity and expression are decreased in response to LPS exposure

**Fig. 3:**
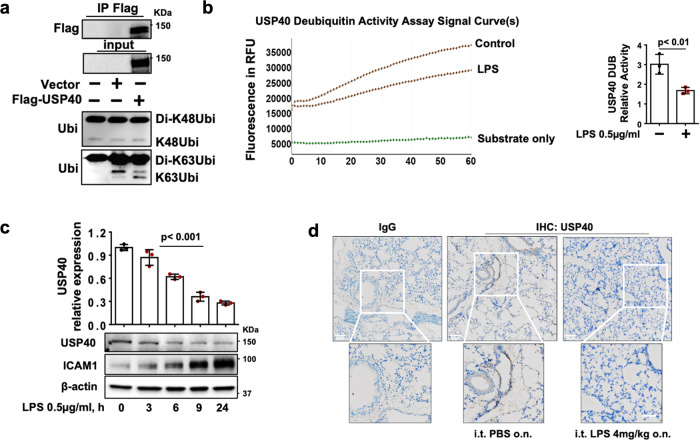
USP40-mediated K63-linked deubiquitination activity and USP40 expression are reduced in response to LPS treatment. **a** HLMVECs were transfected with empty vector or the Flag-USP40 plasmid for 48 h, and Flag-USP40 was immunoprecipitated with an anti-Flag tag antibody, followed by incubation with di-K48-linked ubiquitin (di-K48Ubi) or di-K63-linked ubiquitin (di-K63Ubi). Immunoblot analysis was performed to detect USP40 DUB activity. **b** HLMVECs were transfected with empty vector or the Flag-USP40 plasmid for 48 h, and Flag-USP40 was immunoprecipitated with an anti-Flag tag antibody, followed by incubation with AMC-conjugated ubiquitin (Ub-AMC). USP40 DUB activity was measured by Ub-AMC analysis (*n* = 3). **c** HLMVECs were treated with LPS for 3–24 h. Immunoblot analysis was performed with the indicated antibodies. Fold changes in the USP40/β-actin ratio were analyzed (*n* = 3). ICAM1 expression was used as a positive control for LPS treatment. **d** USP40 immunohistochemical staining in lung tissues from mice with i.t. instillation of PBS or LPS. Scale bars = 50 µm.

To investigate whether USP40 plays a role in the pathogenesis of acute inflammatory diseases, we examined the activity and expression of USP40 in response to LPS. To determine whether USP40 catalyzes the cleavage of K48- and K63-linked polyubiquitin chains, USP40 was immunoprecipitated and then incubated with K48- or K63-linked diubiquitin. As shown in Fig. 3a, USP40 catalyzes the cleavage of K63-linked but not K48-linked ubiquitin chains. This deubiquitinase activity was reduced by LPS treatment in HLMVECs (Fig. 3b). Furthermore, the USP40 abundance was decreased in a time-dependent manner in response to LPS exposure (Fig. 3c). USP40 was highly expressed in vessels in lungs from control mice, but its expression was decreased in LPS-challenged lungs (Fig. 3d). USP40 activity and expression are reduced in response to LPS exposure in cells and murine lungs, suggesting that these reductions contribute to inflammatory responses by increasing EC permeability and inflammation.

### USP40 protects against LPS-induced acute lung injury

**Fig. 4:**
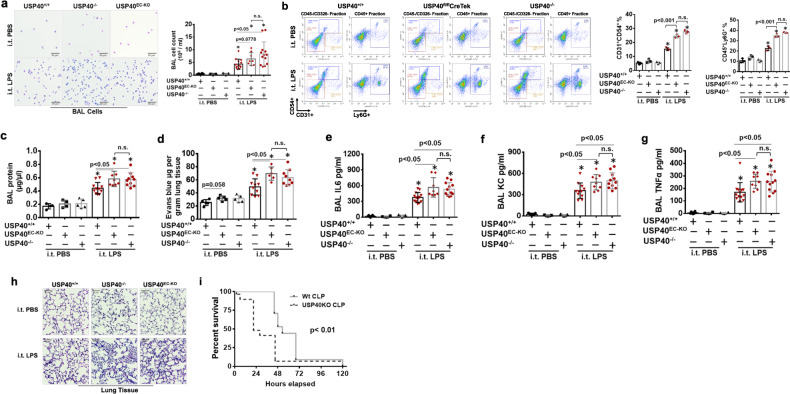
Mice with global USP40 deficiency and EC-specific USP40 deficiency (USP40^EC-KO^) exhibit increased lung injury. USP40^+/+^, USP40^−/-,^ and USP40^EC-KO^ mice were challenged by i.t. instillation of LPS (2 mg/kg) for 24 h. BAL cells were analyzed by cytological staining (**a**), and flow cytometric analysis of cells from LPS-exposed lung tissue was performed (**b**). A gating strategy was used to identify populations expressing the endothelial cell surface marker CD31 (CD45−/CD326−/CD31+) and the neutrophil marker Ly6G (CD45+/Ly6G+). Representative plots showing the percentages of CD54+ endothelial cells and Ly6G+ neutrophils in the lungs of mice treated with PBS or LPS (i.t. instillation, 2 mg/kg, 24 h). Differences among the three groups were compared using one-way ANOVA with Tukey’s multiple comparison test. **c** BAL protein levels were measured. **d** USP40^+/+^, USP40^−/−^, and USP40^EC-KO^ mice were challenged by i.t. instillation of LPS (2 mg/kg), 24 h. Evans blue (EB) was injected into the tail vein 30 min before the mice were sacrificed. Evans blue extravasation in lung tissues was measured. **e**–**g** mIL-6, KC and TNFα concentrations in BAL fluid were measured by ELISA. **h** Lung tissues were subjected to H&E staining. Scale bars = 50 µm. **i** Mortality curve for CLP mice in the USP40^−/−^ (*n* = 16) and wild-type control (Wt, *n* = 15) groups.

**Fig. 5:**
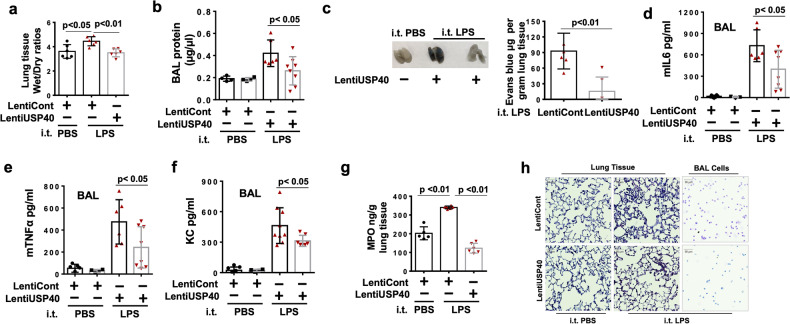
Overexpression of USP40 ameliorates experimental acute lung injury. C57/BL6J mice injected intravenously with lentiviral vector (LentiCont) and lentiviral USP40 (LentiUSP40) were challenged by i.t. instillation of LPS (2 mg/kg) for 24 h. **a**. The wet/dry ratio of lung tissues was measured. **b** BAL protein levels were measured. **c** Evans blue was injected into the tail vein 30 min before the mice were sacrificed. Lung tissues were imaged, and Evans blue extravasation in the lung tissues was measured. **d**–**f** mIL-6, KC and TNFα concentrations in BAL fluid were measured by ELISA. **g** The MPO content in lung tissues was analyzed. **h** Lung tissues and BAL cells were subjected to H&E staining and cytological staining. Scale bars = 50 µm.

To further investigate whether the reductions in USP40 expression and activity contribute to the pathogenesis of acute lung injury, mice with EC-specific USP40 deficiency (USP40^EC-KO^) were generated by crossbreeding USP40floxp/floxp mice with Tek-Cre^1Ywa/J^ or Cdh5-Cre^7Mlia/J^ transgenic mice (Supplementary Figs. 4, 5). Intratracheal administration of LPS increased neutrophil influx into alveolar spaces, and this effect was enhanced in USP40^−/−^ and USP40^EC-KO^ mice (Fig. 4a). Furthermore, lung endothelial cell expression of ICAM1 (CD54) and neutrophil infiltration in LPS-challenged mice were assessed by flow cytometric analysis (Fig. 4b). Depletion of USP40 (USP40^−/−^ and USP40^EC-KO^) significantly increased ICAM1 expression in endothelial cells and neutrophil infiltration in the lungs of mice treated with LPS (i.t. instillation, 2 mg/kg, 24 h). Increased ICAM-1 expression on inflamed endothelial cells enhances leukocyte adhesion and promotes neutrophil transendothelial migration^12^. LPS challenge increased protein levels in bronchoalveolar lavage (BAL) fluid and Evans blue extravasation in lung tissues, indicating that LPS induces microvascular leakage in the lungs. These effects were enhanced in USP40^−/−^ and USP40^EC-KO^ mice (Fig. 4c, d). Depletion of USP40 promoted the LPS-induced increases in the levels of cytokines, including IL-6, KC, and TNFα, in BAL fluid (Fig. 4e–g). Histological staining indicated that both types of USP40 depletion in mice worsened lung injury (Fig. 4h). Considering that Tek-Cre mice exhibit substantial hematopoietic deletion, USP40^*cdh5*-ECKO^ mice were generated, and the role of endothelial USP40 in LPS-induced acute lung injury was confirmed (Supplementary Fig. 6). Furthermore, we evaluated the survival rate of USP40^−/−^ mice compared with that of WT mice after CLP. As shown in Fig. 4i, USP40^−/−^ mice exhibited a significantly lower survival rate. To investigate whether overexpression of USP40 mitigates LPS-induced acute lung injury, a lentiviral vector containing the USP40 gene was administered to mice. Both lung epithelial and endothelial cells exhibited ectopic expression of USP40, but infiltrated neutrophils did not, as shown in Supplementary Fig. 7. Introduction of lenti-USP40 significantly reduced the lung wet/dry ratio (Fig. 5a), BAL protein levels (Fig. 5b), Evans blue extravasation (Fig. 5c), BAL cytokine levels (Fig. 5d–f), the MPO level (Fig. 5g), and neutrophil influx (Fig. 5h) in mouse lungs after i.t. LPS administration. Taken together, these data demonstrate that depletion of USP40 worsens lung injury, while induction of USP40 expression by lentiviral delivery ameliorates lung injury.

### USP40 deubiquitinates and inactivates HSP90β

**Fig. 6:**
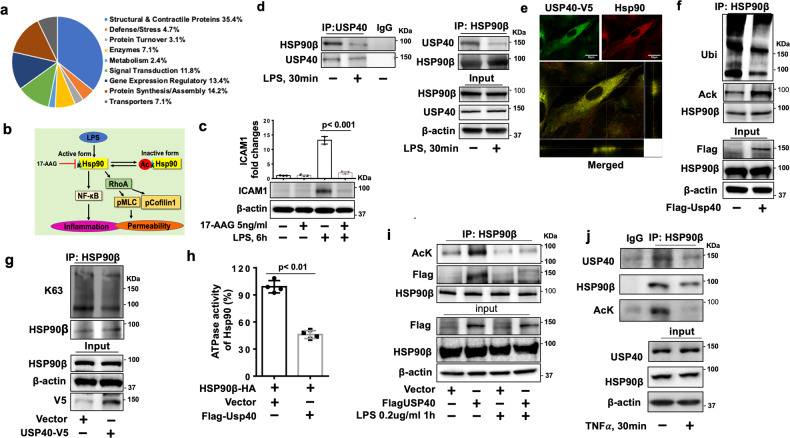
USP40 deubiquitinates HSP90β, resulting in HSP90β hyperacetylation and inactivation. **a** Proteomic profiling of USP40interacting protein complexes in HLMVECs. **b** Schematic diagram showing the role of HSP90 in EC inflammation and hyperpermeability. **c** HLMVECs were treated with 17-AAG prior to stimulation with LPS (0.2 μg/ml) for 6 h. ICAM1 expression was analyzed by immunoblotting. Quantification of ICAM1 expression relative to β-actin expression was performed. **d** HLMVECs were treated with LPS (0.2 μg/ml) for 30 min, and cell lysates were subjected to IP with an anti-USP40 antibody or an anti-HSP90β antibody, followed by immunoblot analysis of USP40 or HSP90β. **e** HLMVECs were transfected with the USP40-V5 plasmid for 48 h. The cells were subjected to immunofluorescence staining with anti-V5 (green) and anti-HSP90β (red) antibodies. Scale bars = 50 µm. **f** HLMVECs were transfected with empty vector or the Flag-USP40 plasmid for 48 h. Denatured proteins in cell lysates were subjected to IP with an anti-HSP90β antibody followed by immunoblotting with anti-ubiquitin or anti-acetylated lysine (AcK) antibodies. **g** HLMVECs were transfected with empty vector or the USP40-V5 plasmid for 48 h, and denatured proteins in cell lysates were subjected to IP with an anti-HSP90β antibody followed by immunoblotting with an anti-K63-linked ubiquitin antibody. **h** HLMVECs were cotransfected with the HSP90β-HA plasmid and empty vector or the Flag-USP40 plasmid for 48 h. HSP90β-HA was immunoprecipitated, and its ATPase activity was measured (*n* = 4). **i** HLMVECs were transfected with empty vector or the Flag-USP40 plasmid for 48 h, followed by LPS treatment for 1 h. Cell lysates were subjected to IP with an antibody against HSP90β followed by immunoblotting with the indicated antibodies. **j** HLMVECs were treated with TNFα (10 ng/ml) for 30 min. Cell lysates were subjected to IP with an antibody against HSP90β followed by immunoblotting with the indicated antibodies.

To further investigate the molecular mechanisms by which USP40 preserves endothelial biological functions, we used an unbiased strategy to identify USP40-interacting proteins. Coimmunoprecipitation (Co-IP) followed by proteomic analysis showed that USP40 interacts with multiple functional proteins (Fig. 6a, Table 1). Among the potential USP40 substrates, HSP90β plays a central role in the regulation of EC dysfunction through inhibition of the NF-κB pathway and Rho-mediated EC stress fiber formation (Fig. 6b). Consistent with previous studies, inhibition of HSP90β attenuated LPS-induced ICAM1 expression on HLMVECs (Fig. 6c). The co-IP and coimmunofluorescence staining experiments confirmed that USP40 interacted with HSP90β (Fig. 6d, e), while this interaction was diminished after LPS or TNFα treatment (Fig. 6d, i, j). Ubiquitination of HSP90β has been reported^23,24^. We found that USP40 reduced the polyubiquitination and increased the lysine acetylation of HSP90β (Fig. 6f). Consistent with data showing that USP40 catalyzes the cleavage of K63-linked ubiquitin chains (Fig. 3a), we found that USP40 catalyzed the cleavage of K63-linked polyubiquitin chains on HSP90β (Fig. 6g). Furthermore, USP40 reduced HSP90β ATPase activity (Fig. 6h). It has been shown that lysine acetylation of HSP90β negatively regulates HSP90β activity^19^. USP40 overexpression increased lysine acetylation of HSP90β (Fig. 6i, j), suggesting that USP40 deubiquitinates HSP90β, resulting in an increase in lysine acetylation of HSP90β, thereby leading to inactivation of HSP90β and preservation of EC integrity. It has been reported that USP40 knockdown disrupts the permeability of glomerular endothelial cells in zebrafish by reducing the nestin level^25^. We also investigated whether nestin plays a role in USP40-mediated biological functions of lung ECs and found that nestin expression was not reduced in HLMVECs with USP40 downregulation or the lungs of USP40^−/−^ mice (Fig. 2g and Supplementary Fig. 2a). Thus, USP40 preserves the biological functions of lung endothelial cells by targeting HSP90β but not nestin.

**Table 1:** The proteins identified in USP40 full-down complex from human lung microvascular endothelial cells.

Protein Name	Accession Number	Molecular Weight
(a) Structural & Contractile Proteins		
Myosin-9 GN = MYH9	P35579	227 kDa
Filamin-A GN = FLNA	P21333	281 kDa
Myosin-10 GN = MYH10	P35580	229 kDa
Alpha-actinin-1 GN = ACTN1	P12814	103 kDa
Plectin GN = PLEC	Q15149	532 kDa
Vimentin GN = VIM	P08670	54 kDa
Actin, cytoplasmic 1 GN = ACTB	P60709	42 kDa
Filamin-B GN = FLNB	O75369	278 kDa
Alpha-actinin-4 GN = ACTN4	O43707	105 kDa
Filamin-C GN = FLNC	Q14315	291 kDa
Unconventional myosin-Id GN = MYO1D	O94832	116 kDa
Supervillin GN = SVIL	O95425	248 kDa
Myosin light polypeptide 6 GN = MYL6	B7Z6Z4	27 kDa
Hornerin GN = HRNR	Q86YZ3	282 kDa
Tubulin alpha-1C chain GN = TUBA1C	F5H5D3	58 kDa
Myosin regulatory light chain 12B GN = MYL12B	O14950	20 kDa
Isoform 4 of Ankycorbin GN = RAI14	Q9P0K7-4	107 kDa
Isoform 4 of LIM domain and actin-binding protein 1 GN = LIMA1	Q9UHB6-4	85 kDa
Tubulin beta chain GN = TUBB	P07437	50 kDa
Drebrin GN = DBN1	Q16643	71 kDa
Isoform 2 of Synaptopodin GN = SYNPO	Q8N3V7-2	96 kDa
Desmoplakin GN = DSP	P15924	332 kDa
Spectrin alpha chain, non-erythrocytic 1 GN = SPTAN1	A6NG51	285 kDa
Cofilin-1 GN = CFL1	P23528	19 kDa
F-actin-capping protein subunit alpha-2 GN = CAPZA2	P47755	33 kDa
Nestin GN = NES	P48681	177 kDa
Filaggrin-2 GN = FLG2	Q5D862	248 kDa
Capping protein (Actin filament) muscle Z-line, beta GN = CAPZB	B1AK87	29 kDa
Isoform 2 of Tropomyosin alpha-3 chain GN = TPM3	P06753-2	29 kDa
DSG1_HUMAN Desmoglein-1 OS=Homo sapiens GN = DSG1	Q02413	114 kDa
Junction plakoglobin GN = JUP	P14923	82 kDa
Gelsolin GN = GSN	F5H1A8	81 kDa
Engulfment and cell motility protein 2 GN = ELMO2	B4DRL5	84 kDa
Cytospin-A GN = SPECC1L	Q69YQ0	125 kDa
Serpin H1 GN = SERPINH1	P50454	46 kDa
Talin-1 GN = TLN1	Q9Y490	270 kDa
Actin-related protein 2/3 complex subunit 4 GN = ARPC4	P59998	20 kDa
Isoform 3 of Unconventional myosin-Ic GN = MYO1C	O00159-3	120 kDa
Actin-related protein 2 GN = ACTR2	P61160	45 kDa
F-actin-capping protein subunit alpha-1 GN = CAPZA1	P52907	33 kDa
Actin-related protein 3 GN = ACTR3	P61158	47 kDa
Isoform 2 of Spectrin beta chain, non-erythrocytic 1 GN = SPTBN1	Q01082-3	251 kDa
Tropomodulin-3 GN = TMOD3	Q9NYL9	40 kDa
Isoform 2 of Protein flightless-1 homolog GN = FLII	Q13045-2	138 kDa
Cytoskeleton-associated protein 4 GN = CKAP4	Q07065	66 kDa
(b) Defense/Stress		
78 kDa glucose-regulated protein GN = HSPA5	P11021	72 kDa
Heat shock protein HSP 90-beta GN = HSP90AB1	P08238	83 kDa
Ataxin-2-like protein GN = ATXN2L	Q8WWM7	113 kDa
Heat shock cognate 71 kDa protein GN = HSPA8	P11142	71 kDa
Endoplasmin GN = HSP90B1	P14625	92 kDa
GTPase IMAP family member 1 GN = GIMA1	Q8WWP7	34 kDa
(c) Protein Turnover		
E3 ubiquitin-protein ligase TRIM21 GN = TRIM21	P19474	54 kDa
LIM domain only protein 7 GN = LMO7	F8WD26	185 kDa
26 S proteasome non-ATPase regulatory subunit 2 GN = PSMD2	Q13200	100 kDa
OTU domain containing 4 GN = OTUD4	G3V0I6	124 kDa
(d) Enzyme		
SCY1-like protein 2 GN = SCYL2	Q6P3W7	104 kDa
Tyrosine-protein kinase JAK1 GN = JAK1	P23458	133 kDa
Protein phosphatase 1B GN = PPM1B	O75688	53 kDa
Protein arginine N-methyltransferase 5 GN = PRMT5	G3V5W5	68 kDa
Peroxiredoxin-1 GN = PRDX1	Q06830	22 kDa
Protein-glutamine gamma-glutamyltransferase 2 GN = TGM2	P21980	77 kDa
Serine/threonine-protein kinase 38 GN = STK38	Q15208	54 kDa
Peptidyl-prolyl cis-trans isomerase A GN = PPIA	P62937	18 kDa
Serine/threonine-protein kinase 38-like GN = STK38L	Q9Y2H1	54 kDa
(e) Metabolism		
Pyruvate kinase PKM GN = PKM	P14618	58 kDa
6-phosphofructo-2-kinase GN = PFKFB3	F2Z2I2	53 kDa
Glyceraldehyde-3-phosphate dehydrogenase GN = GAPDH	P04406	36 kDa
(f) Signal Transduction		
Interferon-induced GTP-binding protein Mx2 GN = MX2	P20592	82 kDa
Annexin A2 GN = ANXA2	P07355	39 kDa
Dedicator of cytokinesis protein 4 GN = DOCK4	Q8N1I0	225 kDa
Thyroid hormone receptor-associated protein 3 GN = THRAP3	Q9Y2W1	109 kDa
Myosin phosphatase Rho-interacting protein GN = MPRIP	Q6WCQ1	117 kDa
Protein phosphatase 1 regulatory subunit 12 A GN = PPP1R12A	O14974	115 kDa
Isoform 12 of Sorbin and SH3 domain-containing protein 2 GN = SORBS2	O94875-12	81 kDa
Neurabin-2 GN = PPP1R9B	Q96SB3	89 kDa
Zinc finger protein 185 GN = ZNF185	O15231	74 kDa
Plasminogen activator inhibitor 1 GN = SERPINE1	P05121	45 kDa
Interferon-induced GTP-binding protein Mx1 GN = MX1	P20591	76 kDa
Isoform 2 of Leucine zipper protein 1 GN = LUZP1	Q86V48-2	115 kDa
Thrombospondin-1 GN = THBS1	P07996	129 kDa
Zyxin GN = ZYX	B4DQR8	52 kDa
Glia maturation factor beta GN = GMFB	G3V4P8	18 kDa
(g) Gene Expression Regulatory		
Histone H4 OS=Homo sapiens GN = HIST1H4A	P62805	11 kDa
Elongation factor 2 GN = EEF2	P13639	95 kDa
Nuclear fragile X mental retardation-interacting protein 2 GN = NUFIP2	Q7Z417	76 kDa
Histone H2B type 3-B GN = HIST3H2BB	Q8N257	14 kDa
Histone H2A type 2-C GN = HIST2H2AC	Q16777	14 kDa
ATP-dependent RNA helicase DDX3X GN = DDX3X	O00571	73 kDa
Heterogeneous nuclear ribonucleoprotein U GN = HNRNPU	Q00839	91 kDa
Isoform 2 of Highly divergent homeobox GN = HDX	Q7Z353-2	70 kDa
Polymerase I and transcript release factor GN = PTRF	Q6NZI2	43 kDa
U4/U6 small nuclear ribonucleoprotein Prp31 GN = PRPF31	Q8WWY3	55 kDa
Isoform 2 of RNA-binding protein 10 GN = RBM10	P98175-2	103 kDa
Splicing factor 3B subunit 3 GN = SF3B3	Q15393	136 kDa
Bcl-2-associated transcription factor 1 GN = BCLAF1	Q9NYF8	106 kDa
Eukaryotic initiation factor 4A-I GN = EIF4A1	P60842	46 kDa
Methylosome protein 50 GN = WDR77	Q9BQA1	37 kDa
Non-POU domain-containing octamer-binding protein GN = NONO	Q15233 ( + 1)	54 kDa
Heterogeneous nuclear ribonucleoprotein H2 GN = HNRNPH2	P55795	49 kDa
(h) Protein Synthesis/Assembly		
Ubiquitin-like protein ISG15 GN = ISG15	P05161	18 kDa
40 S ribosomal protein S18 GN = RPS18	P62269	18 kDa
40 S ribosomal protein S16 GN = RPS16	P62249	16 kDa
Putative elongation factor 1-alpha-like 3 GN = EEF1A1P5	Q5VTE0	50 kDa
40 S ribosomal protein S25 GN = RPS25	P62851	14 kDa
60 S ribosomal protein L11 GN = RPL11	P62913	20 kDa
40 S ribosomal protein S15a GN = RPS15A	P62244	15 kDa
60 S ribosomal protein L28 GN = RPL28	H0YMF4	12 kDa
40 S ribosomal protein S20 GN = RPS20	P60866	13 kDa
40 S ribosomal protein S9 GN = RPS9	P46781	23 kDa
60 S ribosomal protein L12 GN = RPL12	P30050	18 kDa
Heterogeneous nuclear ribonucleoprotein K GN = HNRNPK	P61978	51 kDa
60 S ribosomal protein L23 GN = RPL23	P62829	15 kDa
Isoform 4 of 40 S ribosomal protein S24 GN = RPS24	P62847-4	32 kDa
40 S ribosomal protein S14 GN = RPS14	P62263	16 kDa
Eukaryotic translation initiation factor 4BGN = EIF4B	E7EX17	70 kDa
40 S ribosomal protein S27 GN = RPS27L	H0YMV8	11 kDa
40 S ribosomal protein S13 GN = RPS13	P62277	17 kDa
(i) Transporter		
Caveolin-1 GN = CAV1	Q03135	20 kDa
ADP-ribosylation factor 3 GN = ARF3	P61204	21 kDa
Major vault protein GN = MVP	Q14764	99 kDa
Erythrocyte band 7 integral membrane protein GN = STOM	B4E2V5	26 kDa
Clathrin heavy chain 1 GN = CLTC	Q00610	192 kDa
ATP synthase subunit alpha, mitochondrial GN = ATP5A1	P25705	60 kDa
ADP-ribosylation factor 4 GN = ARF4	P18085	21 kDa
EH domain-containing protein 4 GN = EHD4	Q9H223	61 kDa
ADP/ATP translocase 2 GN = SLC25A5	P05141	33 kDa

### USP40 docking site and acetylation site in HSP90β

**Fig. 7:**
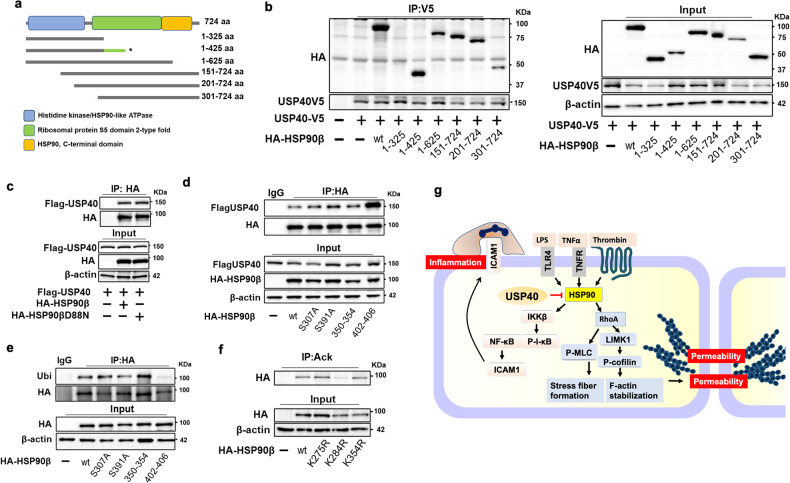
Analysis of the USP40 and Hsp90β interaction and identification of the acetylation site in HSP90β. **a** Schematic diagram of HSP90β deletion mutants. The structure of HSP90β consists of an N-terminal ATPase domain, a ribosomal protein S5 domain 2, and a C-terminal domain. The HSP90β C-terminal and N-terminal deletion mutants were generated by site-specific mutagenesis, and the corresponding plasmids were constructed. **b** HEK293 cells were cotransfected with USP40-V5 and HA-tagged N- or C-terminal deletion mutants of HSP90β for 48 h. The USP40 binding site in HSP90β was identified by co-IP of USP40-V5 using an anti-V5 antibody followed by immunoblotting with an antibody against the HA tag. **c** HEK293 cells were cotransfected with Flag-USP40 and vector or HA-tagged wild-type HSP90β or the dominant-negative (D88N) HSP90β mutant for 48 h. Cell lysates were subjected to IP with an antibody against HA followed by immunoblotting with the indicated antibodies. **d** HEK293 cells were cotransfected with Flag-USP40 and vector or HA-tagged wild type (wt) HSP90β or HSP90β mutants (S307A, S391A, ∆350–354, ∆402–406) for 48 h. Cell lysates were subjected to IP with an antibody against the HA tag followed by immunoblotting with the indicated antibodies. **e** HEK293 cells were transfected with HA-tagged wild type (wt) Hsp90β or Hsp90β mutants (S307A, S391A, ∆350–354, ∆402–406) for 48 h. The ubiquitination of HSP90 was examined by modified IP (under denaturing conditions) with an anti-HA antibody, followed by immunoblotting with antibodies against ubiquitin (Ubi) and HA. **f** HEK293 cells were transfected with HA-tagged wild-type (wt) HSP90β or HSP90β mutants (K275R, K284R, and K354R) for 48 h. Cell lysates were subjected to IP with an antibody against acetylated lysine (AcK) followed by immunoblotting with the indicated antibodies. **g** USP40 preserves EC integrity by deubiquitinating and inactivating HSP90β, resulting in reductions in inflammatory stimulus-induced EC inflammation and hyperpermeability. Schematic diagram showing HSP90β-mediated NF-κB pathway activation and cytoskeletal rearrangement in ECs.

To identify a specific HSP90 motif interacting with USP40, HSP90 C-terminal and N-terminal deletion mutants were generated by mutagenesis, and the corresponding plasmids were constructed (Fig. 7a). Only the N-terminal fragment (1-325 aa) of HSP90β was unable to bind to USP40, suggesting that the USP40 binding site is located between residues 325 and 425 of HSP90β (Fig. 7b). Independent of HSP90 activity, wild-type HSP90 and the dominant-negative mutant (HSP90βD88N) had similar binding affinities for USP40 (Fig. 7c). To determine the mechanism of USP40-mediated deubiquitination of Hsp90β, several constructs with mutations in the region between aa 300 and 425 were generated by site-specific mutagenesis. HSP90β^∆402-406^ was found to demonstrate enhanced binding to USP40 (Fig. 7d), suggesting that the region between residues 402 and 406 is most likely also involved in the interaction but inhibits binding. Furthermore, expression of HSP90^∆402-406^ significantly reduced the monoubiquitination of HSP90β (Fig. 7e), possibly due to the high binding affinity of this mutant for USP40. These data indicate that targeting the ^402^KVIRK^406^ sequence with a small molecule may attenuate proinflammatory responses and pulmonary EC barrier disruption by enhancing the association of Hsp90β with USP40. Furthermore, we confirmed that K284 is an acetylation site in Hsp90β (Fig. 7f), consistent with the previously reported K294 in Hsp90α^34^.

## Discussion

Ubiquitination is involved in inflammatory responses and cell‒cell junctions^22^. Our previous studies showed that E3 ligases such as FBXL19, Nedd4L, and FBXL2 exhibit anti-inflammatory properties^35–38^, while DUBs, such as USP11 and USP14, play proinflammatory roles^38,39^. In this study, we focused on the role of a DUB in EC inflammation and barrier dysfunction. We revealed that USP40 preserves EC function by deubiquitinating and inactivating HSP90β. USP40, a newly recognized DUB, was reported to regulate glomerular permeability in zebrafish by mediating nestin expression^25^. A study from the same group further demonstrated that depletion of Usp40 in zebrafish disrupted glomerulogenesis, but surprisingly, Usp40KO mice showed a normal kidney phenotype. Additionally, this group showed that in contrast to the reduction in nestin expression in Usp40-depleted zebrafish, nestin was upregulated in the glomeruli of Usp40KO mice^26^. We confirmed that the nestin level was not changed in USP40-deficient HLMVECs or the lungs of USP40KO mice (Fig. 2g and Supplementary Fig. 2a), indicating that the mechanism by which USP40 preserves lung EC barrier integrity is not mediated through nestin. Here, we reveal a new mechanism of regulation of HSP90β deubiquitination and inactivation by USP40.

HSP90 is a critical modulator of cell signaling and has been shown to play a detrimental role in EC integrity^16^. Inhibition of HSP90 protects lung EC integrity by attenuating NF-κB pathway activity and cytoskeletal rearrangement^17,18^. USP40 deubiquitinates HSP90β, resulting in the inactivation of HSP90β in ECs, suggesting that USP40 protects EC integrity by blocking HSP90β-mediated EC dysfunction. This is the first study to identify a DUB that regulates both the inflammation and barrier function of ECs. USP40 has been shown to cleave K48-linked polyubiquitin chains on c-FLIPL in lung cancer cells^40^. In ECs, USP40 does not catalyze the cleavage of K48-linked polyubiquitin chains. However, USP40 catalyzes the cleavage of K63-linked polyubiquitin chains on HSP90β. Unlike other reported substrates, USP40 regulates HSP90β deubiquitination without altering HSP90β stability. In general, K63-linked polyubiquitination regulates protein trafficking, protein‒protein interactions, and enzyme activity, while K48-linked polyubiquitination promotes proteasomal protein degradation^21,22^. HSP90β can undergo K48- and K63-linked polyubiquitination mediated by a ubiquitin E3 ligase, CHIP^23^. Hectd1, another ubiquitin E3 ligase, mediates K63-linked polyubiquitination, leading to the cytosolic localization of HSP90 and a reduction in HSP90 secretion^24^. Hyperacetylation of HSP90β reduces its chaperone activity^19^. In this study, we reveal that USP40 increases the acetylation and decreases the activation of HSP90β. Consistent with a previous report, lysine 284 is an acetylation site in Hsp90β^34^. Of particular interest is determining whether deubiquitination by USP40 modulates the interaction between HSP90 and its lysine acetyltransferase or deacetylase. Taken together with findings showing that USP40 deubiquitinates HSP90β, these data suggest that USP40 inactivates HSP90β by modulating its ubiquitination and acetylation.

The detrimental effect of HSP90 activity in ECs has been well reported^17,41^. Inhibition of HSP90 suppresses NF-κB signaling and prevents the nuclear translocation of NF-κB, thereby diminishing inflammation^17,42^. In ECs, HSP90 inhibitors prevent LPS-induced endothelial barrier dysfunction by attenuating RhoA-mediated phosphorylation of MLC^16^. HSP90 inhibition protects the brain microvascular endothelium against oxidative stress^43^. A large amount of evidence indicates that inhibition of HSP90 is a potential therapeutic strategy for EC dysfunction-related inflammatory diseases^16,18,41,43^. HSP90 has been shown to associate with DUBs, such as USP19 and USP50^44,45^. HSP90 regulates the DUB activity of USP19 and promotes USP50-mediated Wee1 stability^45^; however, a DUB for HSP90 has not been reported. This is the first report to reveal that USP40 deubiquitinates and inactivates HSP90. Consistent with findings indicating that inhibition of HSP90 diminishes EC inflammation and barrier dysfunction, USP40 protects lung ECs against inflammatory stimulus-induced inflammatory responses and barrier disruption in vitro and in vivo. Nestin is a type VI intermediate filament protein and is a developmentally regulated protein^46^. It seems that nestin is not involved in EC inflammation and barrier function. Nestin is considered to play a role in the formation of the cytoskeleton in newly formed ECs^46^; thus, USP40 may play a role in vascular regeneration during lung repair and remodeling.

We showed that the USP40 level was reduced in response to endotoxin exposure. USP40 (USP40^−/−^ and USP40^EC-KO^) deficiency significantly exacerbated lung injury. Consistent with a previous study^40^, no apparent abnormal phenotypes were observed in USP40KO or USP40 EC-KO mice. We believe that the pathological phenotypes resulting from USP40 deletion in this study are not secondary to preexisting vascular and nonvascular changes during embryogenesis and adulthood. We employed mice with endothelium-specific deletion of USP40 to determine the role of endothelial USP40. Although Tek-Cre mice have been reported to show at least partial Cre recombinase activity in cells of the hematopoietic lineage, via the TgTek-cre^1Ywa/J^ allele, a small number of circulating cells have been reported to be Cre positive in adult mice^47^. Thus, a portion of immune cells may also lack USP40 expression in the Tek-Cre system. This might be due to Cre leakiness in the Tek-Cre system, as there were no differences in BAL cell counts, vascular leakage, or BAL cytokine levels between USP40KO (global knockout) mice and USP40^EC-KO^ mice with USP40 deficiency driven by Tek-Cre (Fig. 4). USP40^*cdh5*-ECKO^ mice were generated by breeding USP40floxp/floxp mice with Cdh5-cre^7Mlia/J^ transgenic mice. As shown in Supplementary Fig. 5c and 5d, USP40 excision events in blood cells were not detected in the Cdh5-Cre system. The increased lung injury in mice with EC-specific USP40 deficiency (USP40^*cdh5*-ECKO^) confirmed the protective effects of USP40 in ECs (Supplementary Fig. 6).

Taken together, this study shows that USP40 deubiquitinates HSP90β, thereby inhibiting its activity by increasing its acetylation (Fig. 7g). We reveal a new molecular mechanism for the regulation of posttranslational modification and activation of HSP90β. HSP90 inhibitors have emerged as potential anti-inflammatory drugs. Understanding the molecular regulation of HSP90β activity by USP40 is expected to lead to the development of new drugs to target HSP90. USP40 activity and protein expression are suppressed under inflammatory conditions, suggesting that these reductions contribute to the pathogenesis of acute lung injury. The molecular regulation of USP40 expression and activity is anticipated to be a new focus for understanding the processes of inflammatory diseases and developing new therapeutic strategies for acute lung injury and sepsis.

### Supplementary information

Supplemental figures

## Data Availability

All data and study materials that support the findings of this study are available from the corresponding authors upon reasonable request.
