# Supplementary material for: The deubiquitinase USP40 preserves endothelial integrity by targeting the heat shock protein HSP90β
Source: Exp Mol Med. 2024 Feb 2;56(2):395–407. doi: 10.1038/s12276-024-01160-y (PMC10907362; doi:10.1038/s12276-024-01160-y)
Supplement: Supplementary file 1 — Supplemental figures [file 12276_2024_1160_MOESM1_ESM.pdf]

Supplementary figures

# The deubiquitinase USP40 preserves endothelial integrity by targeting the heat shock protein HSP90β

Jiaxing Miao [1], Lian Li [1], Nargis Shaheen [1], Jianxin Wei [2], Anastasia M Jacko [2], Prithu Sundd [2], Sarah J Taleb [1], Rama K Mallampalli [3], Yutong Zhao [1,3], Jing Zhao [1,3, ¶]

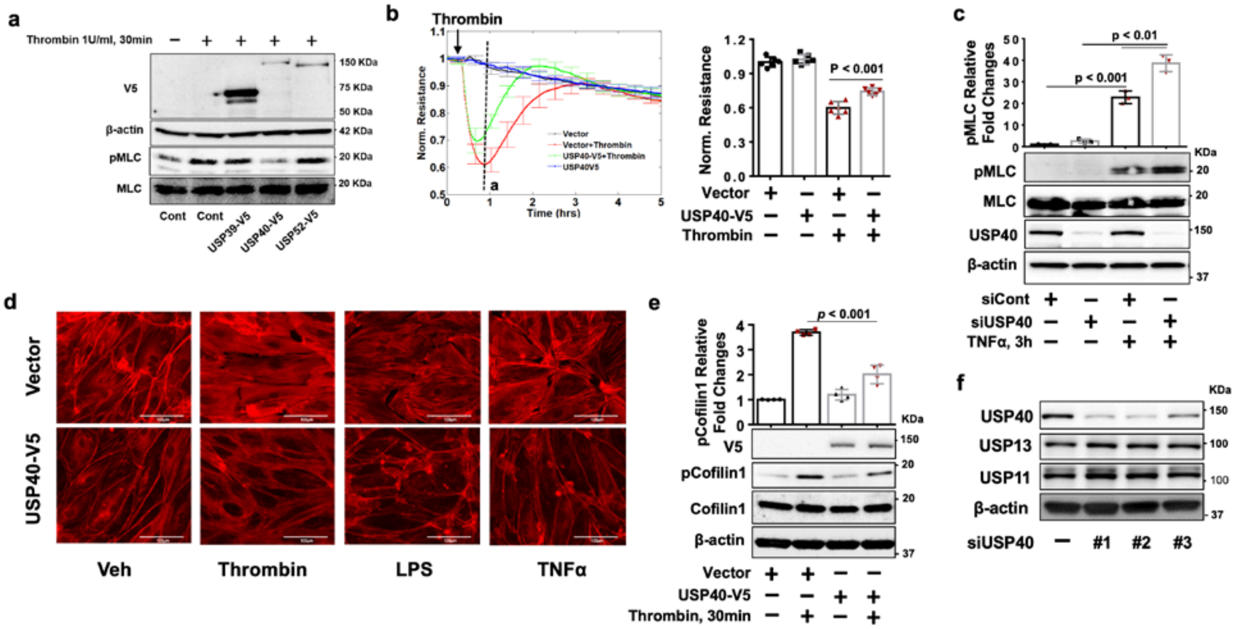


**Supplementary Fig. 1. a.** HLMVECs were transfected with different V5-tagged DUB plasmids for 48 h, and then treated with thrombin 1U/ml for 30 min. Cell lysates were analyzed by immunoblotting with indicated antibodies. **b.** HLMVECs were transfected with empty vector or USP40-V5 plasmid for 48 h, and cells were treated with thrombin (1 U/ml). TEER was measured by ECIS system. Normalized resistance was recorded at 4,000 Hz. Each line represents the mean ± SEM in the specified time points. The electrical resistance at indicated time point (a) was quantified and statistical analysis was performed (right). **c.** HLMVECs were transfected with control siRNA or USP40 siRNA for 72 h, and then were treated with TNFα (10 ng/ml) for 3 h. Cell lysates were analyzed by immunoblotting with indicated antibodies. Fold changes of p-MLC/MLC ratio were analyzed (n=3). **d.** HLMVECs grown on glass-bottom dishes were transfected with empty vector or USP40-V5 plasmid for 48 h, and cells were treated with thrombin (1 U/ml, 30 minutes), LPS (0.2 μg/l, 3 h), or TNFα (5 ng/ml, 3 h). F-actin and stress fibers were stained with fluorescent phalloidin. Scale bars = 100 μm. **e.** HLMVECs were transfected with empty vector or USP40-V5 plasmid for 48 h, and cells were treated with thrombin (1 U/ml) for 30 minutes. Cell lysates were analyzed by immunoblotting with indicated antibodies. Fold changes of pCofilin 1 / Cofilin 1 were analyzed (n=3). **f.** HLMVECs were transfected with control siRNA or USP40 siRNA for 72 h. Cell lysates were analyzed by immunoblotting with indicated antibodies.

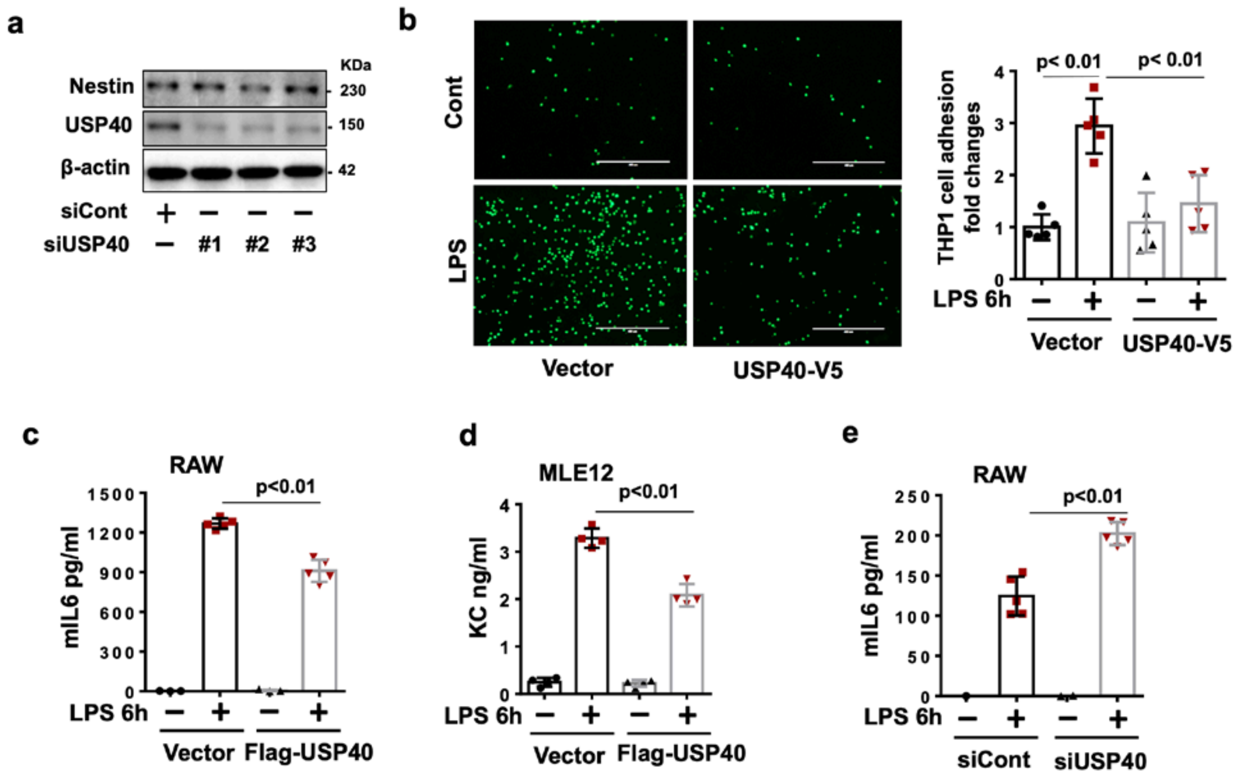


**Supplementary Fig. 2. a.** HLMVECs were transfected with control siRNA or USP40 siRNA for 72 h. Cell lysates were analyzed by immunoblotting with indicated antibodies. **b.** HLMVECs were transfected with empty vector or USP40-V5 plasmid for 48 h. Cells were treated with LPS (0.2 μg/ml) for 6 h, and then fluorescence-labeled THP-1 cell adhesion to HLMVECs were measured by a fluorescence microscope and quantification. **c.** RAW264.7 cells were transfected with empty vector or Flag-USP40 plasmid for 48 h, and then were treated with LPS. mIL-6 in media were measured by ELISA assay. **d.** MLE12 cells were transfected with empty vector or Flag-USP40 plasmid for 48 h, and then were treated with LPS for 16 h. KC in media were measured by ELISA assay. **e.** RAW264.7 cells were transfected with control siRNA or mouse USP40 siRNA for 72 h, and then were treated with LPS for 16 h. mIL-6 in media were measured by ELISA assay.

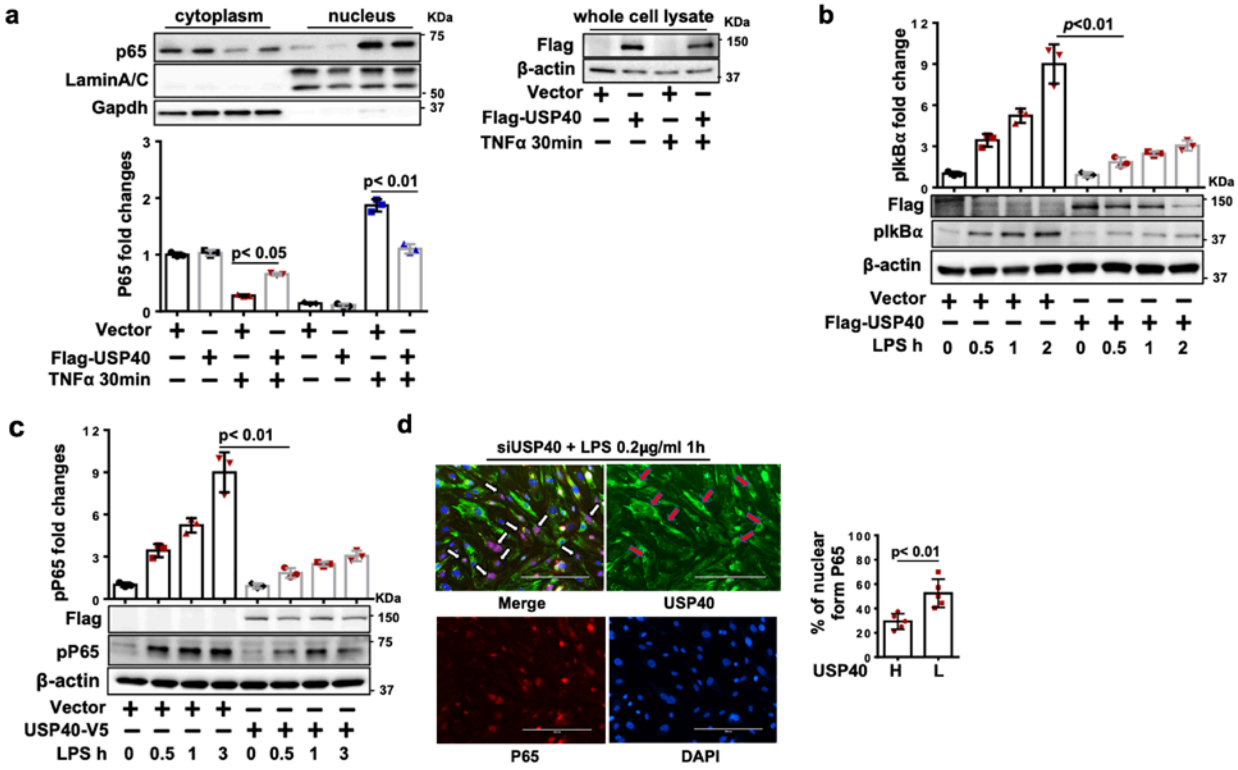


**Supplementary Fig. 3. USP40 reduces NF-κB pathway. a.** HLMVECs were transfected with empty vector or Flag-USP40 plasmid for 48 h, then treated with TNFα for 30 minutes. The cytoplasm and nuclear fractions were isolated and analyzed by immunoblotting. Fold changes of p65 were analyzed (n=3). **b.** HLMVECs were transfected with empty vector or Flag-USP40 plasmid for 48 h, then treated with LPS for 0.5-2 h. Immunoblotting analysis was performed with indicated antibodies. Fold changes of pIκBα/β-actin ratio was analyzed (n=3). Significant differences ($p<0.01$) between the two groups as determined by two-way ANOVA. **c**. HLMVECs were transfected with empty vector or Flag-USP40 plasmid for 48 h, followed by treatment with LPS for 0.5-3 h. Immunoblotting analysis was performed with indicated antibodies. Fold changes of pP65/β-actin ratio was analyzed (n=3). Significant differences ($p<0.01$) between the two groups as determined by two-way ANOVA. **d**. HLMVECs were transfected with control siRNA or USP40 siRNA for 72 h, then treated with LPS 0.2 μg/ml for 1h. Cells were immunofluorescence stained with a USP40 antibody (green) and a P65 antibody (red). Nuclei were stained with DAPI (blue). Red arrows indicate the cells with USP40 high expression and white arrows indicates USP40 low expression. Scale bars = 100 μm. Quantification of percentage of nuclear P65 was analyzed.

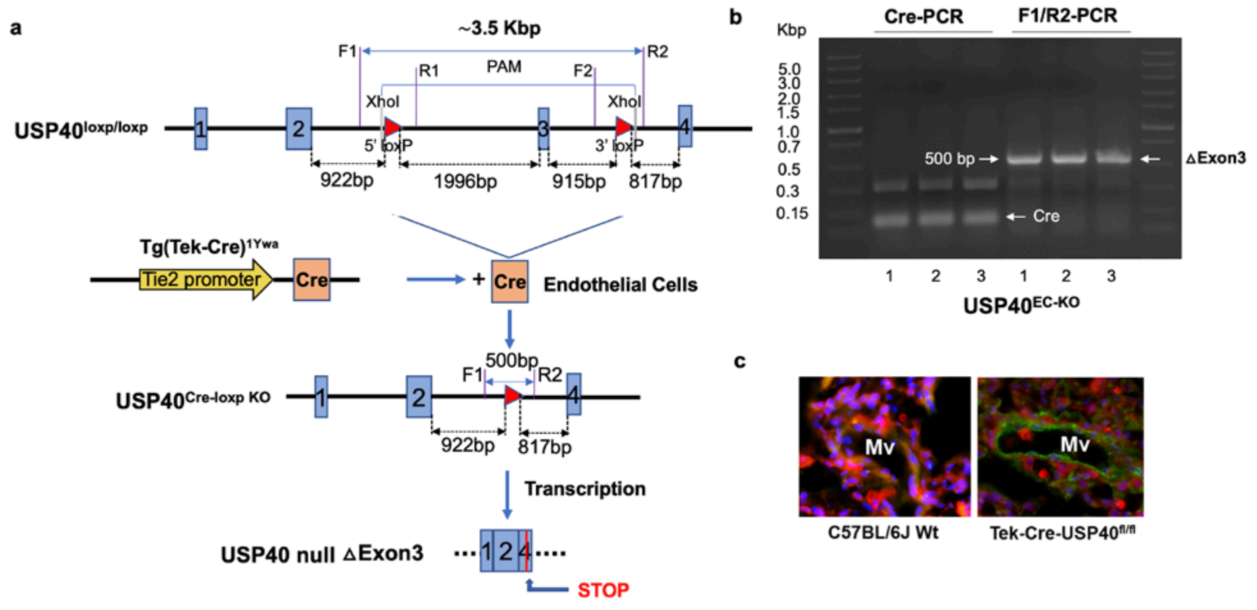


**Supplementary Fig. 4. a.** Scheme shows the strategy to generate USP40Loxp mice. Breeding USP40$^{floxp/floxp}$ mice with Tek-Cre mice generate Tek-Cre-USP40$^{fl/fl}$ (USP40$^{EC-KO}$) mice as described in the methods. 5' and 3' loxp with XhoI were inserted at the upstream and downstream of the Exon 3 of Usp40, respectively. USP40 EC knockout mice were generated by crossing with Tie-Cre transgenic mice (B6.Cg-TgTek-Cre$^{1Ywa/J}$, Jackson Laboratory). Exon 3 of Usp40 was flanked by the two loxp sites in endothelial cells. Following deletion of exon 3 (deleting amino acids 66 to 89), splicing of exon 2 into 4 results in a frameshift leading (after 110 bp) to a premature stop codon in exon 4. **b**. PCR screening of Cre-lox-mediated Usp40 excision events (ΔExon 3) in mouse genome. Cre-PCR lane 1 to 3 (Cre Forward: GCGGTCTGGCAGTAAAAACTATC and Cre Reverse: GTGAAACAGCATTGCTGTCACTT; Internal Positive Control Forward: CTAGGCCACAGAATTGAAAGATCT and Internal Positive Control Reverse: GTAGGTGGAAATTCTAGCATCATCC) and Usp40 F1/R2 PCR (F1 Forward: GCCCCAGACCTTGATAGGAA and R2 Reverse: GCCTCTGAGTGAGATTCCAGC) lane 1 to 3 excision event detection (500bp for deleted allele). **c**. Lung tissues were co-immunostaining with USP40 (Red) and CD31 (Green) antibodies. Mv, micro vessel.

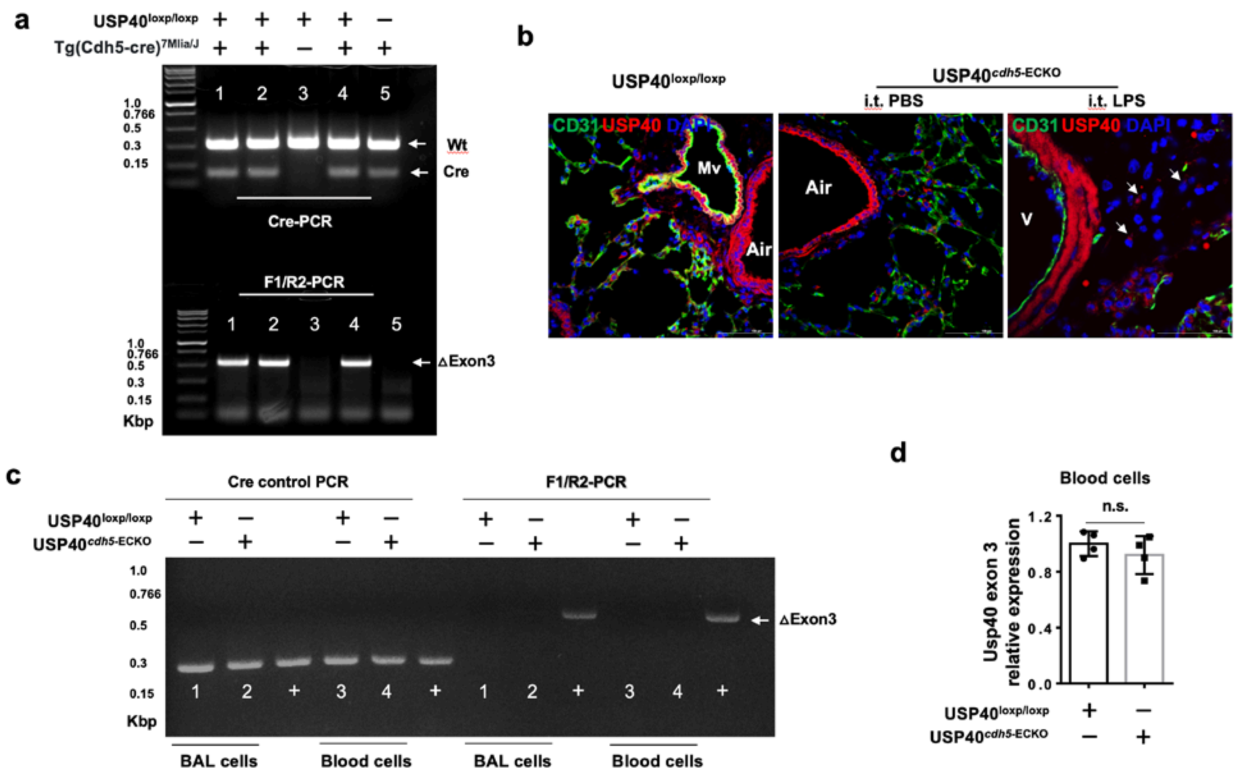


**Supplementary Fig. 5.** Breeding USP40$^{floxp/floxp}$ mice with Cdh5-Cre transgenic mice (B6.Cg-Tg$^{Cdh5\text{-}cre7Mlia/J}$, Jackson Laboratory) to generate Cdh5-Cre-USP40$^{fl/fl}$ (USP40$^{\mathit{cdh5}\text{-}ECKO}$) mice as described in the methods. **a.** PCR screening of Cre-lox-mediated Usp40 excision events in mouse genome with Cre-PCR and Usp40 F1/R2 primers. Mice # 1, 2, and 4 were generated by breeding USP40$^{floxp/floxp}$ mice with Cdh5-Cre transgenic mice with Exon 3 deletion allele (500bp). **b**. Lung tissues were co-immunostaining with USP40 (Red) and CD31 (Green) antibodies. Mv, micro vessel. V, vessel. Air, airway. Arrows indicate neutrophils with USP40 expression. **c.** USP40$^{floxp/floxp}$ and USP40$^{\mathit{cdh5}\text{-}ECKO}$ mice were intratracheally administrated with 2 mg/kg LPS for 24 h. BAL (Lane 1 and 2) and blood cells (Lane 3 and 4) were collected for PCR screening of Cre-lox-mediated Usp40 excision events with Cre control PCR (Internal positive control primers as shown in S4) and Usp40 F1/R2 PCR. +, Usp40 exon3 deletion (ΔExon 3) positive control. Cre-lox-mediated Usp40 excision events were not detected in BAL and circulating cells. **d**. Usp40 exon 3 expression were determined by real time PCR (Usp40 exon 3 Forward: TGGCACCATTGTGAACCAGA and Usp40 exon 3 Reverse: CCACAGTGGTACAGGTTGTCA) in blood cells of i.t. LPS (2 mg/kg, 24 h) treated USP40$^{floxp/floxp}$ and USP40$^{\mathit{cdh5}\text{-}ECKO}$ mice.

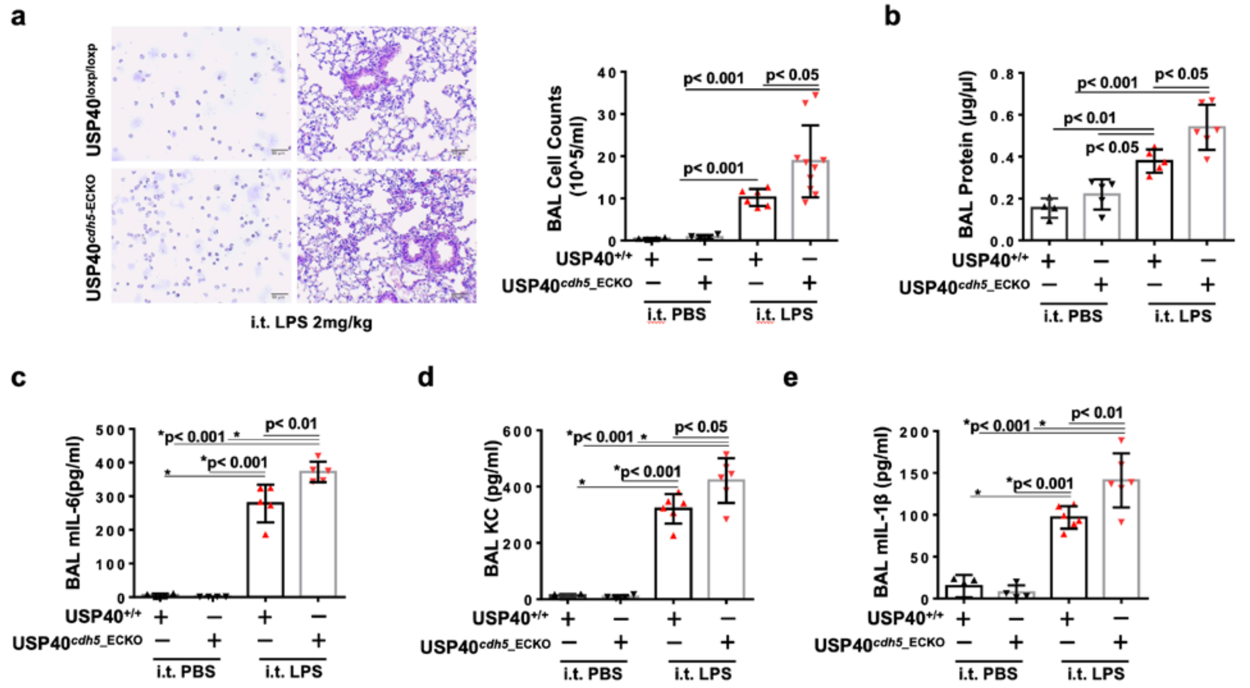


**Supplementary Fig. 6. USP40 EC specific deficient mice (USP40$^{cdh5\text{-ECKO}}$) increase lung injury.** USP40$^{+/+}$ and USP40$^{cdh5\text{-ECKO}}$ mice were challenged with i.t. LPS 2 mg/kg for 24 h. **a**. BAL cells were analyzed by cytology staining and lung tissues were subjected to H&E staining. Scale bars = 50 µm. **b.** BAL protein levels were measured. **c-e.** mIL-6, KC, and IL-1β levels in BAL were measured by ELISA. Differences among the two groups were compared using one-way ANOVA with Turkey's multiple comparisons.

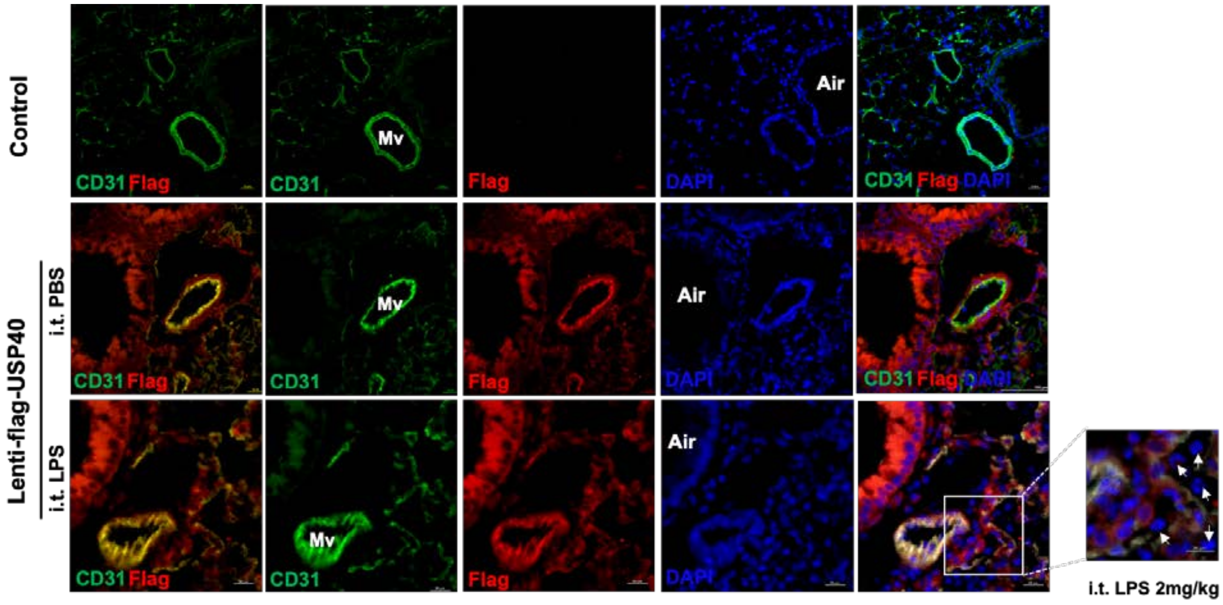


**Supplementary Fig. 7.** C57/BL6J mice infected intravenously with lentivirus control and flag-USP40 for a week were challenged with i.t. LPS 2mg/kg for 24 h. Lung tissues were co-immunostaining with Flag (Red) and CD31 (Green) antibodies. Mv, micro vessel. Air, airway. Arrows indicate neutrophils without flag-USP40 overexpression.
